# Retraction Note: Identification of protein kinase inhibitors to reprogram breast cancer cells

**DOI:** 10.1038/s41419-022-05311-9

**Published:** 2022-10-07

**Authors:** Jie Yuan, Fan Zhang, Meng You, Qin Yang

**Affiliations:** 1 Cancer Biology Division, Department of Radiation Oncology, Washington University School of Medicine, Saint Louis, MO 63108 USA; 2 Medical Center of Stomatology, The First Affiliated Hospital of Jinan University, 510630 Guangzhou, China; 3 School of Stomatology, Jinan University, 510630 Guangzhou, China

Retraction to: *Cell Death and Disease* 10.1038/s41419-018-1002-2, published online 11 September 2018

The Editor-in-Chief has retracted this article. An investigation by the Office of the Vice Chancellor for Research at Washington University in St. Louis concluded that:

- Figure panels 1A, B, and C, labeled as cell line MDA-MB-468, appeared to be falsified and/or fabricated, and were also used in multiple NIH grant applications labeled as breast cancer cell line 4T1 or brain cancer cell line U118.
- Photos in figure panels 2A and 2C, labeled as breast cancer cell line MDA-MB-468, appeared to be falsified, and were also used in multiple NIH grant applications labeled as cell lines 4T1 or BT20.
- Figure panel 2B, labeled as breast cancer cell line MDA-MB-468, appeared to be falsified and/or fabricated, and was also labeled as brain cancer cell line GBM U118 in another publication by the same authors [1].
- Figure 4C, labeled as iFLs from breast cancer cell line MDA-MB-468 in the publication, appeared to be falsified and/or fabricated, and was also used in [1] labeled as iN cells.
- An additional concern identified as part of the investigation is the extensive overlap between this study and another article by the same authors [1].

The authors have not responded to any correspondence from the editor or publisher about this retraction.
